# Co-Exposure to Lunasin and Other Drugs as a Potential Chemopreventive Strategy Against Breast and Colon Cancers: A Review

**DOI:** 10.3390/ijms27136079

**Published:** 2026-07-07

**Authors:** Aleksandra Janiak, Agnieszka Kaufman-Szymczyk, Katarzyna Lubecka-Gajewska

**Affiliations:** Department of Biomedical Chemistry, Faculty of Health Sciences, Medical University of Lodz, 92-215 Lodz, Poland; agnieszka.kaufman-szymczyk@umed.lodz.pl (A.K.-S.); katarzyna.lubecka@umed.lodz.pl (K.L.-G.)

**Keywords:** lunasin, bioactive peptide, soy peptide, chemoprevention, adjunct, breast cancer, colon cancer

## Abstract

More than 20 years after the discovery of lunasin, a clear shift in lunasin research is observable—from an initial focus on its direct in vitro anticancer effects toward strategies aimed at improving its bioavailability and repositioning it as a potential adjunct in cancer therapy. Lunasin, a soy-derived bioactive peptide, has been extensively studied for its antineoplastic properties. However, its limited oral bioavailability restrains its efficacy in clinical trials. Therefore, recent research on lunasin points towards the possibility of using it as an adjunct in cancer treatment, rather than as a stand-alone nutraceutical in humans. In preclinical models, in vitro and in vivo, lunasin can enhance the effects of standard anticancer drugs in breast and colon cancers. Research suggests that lunasin can potentiate the effects of drugs, such as tamoxifen, aspirin, cisplatin, and oxaliplatin, by sensitizing cancer cells to apoptosis, modulating cell cycle progression, reducing metastatic potential, and attenuating drug-resistance pathways, including PI3K/Akt, FAK/MAPK1/NF-κB, and integrin-mediated signaling. In combination with those drugs, lunasin exerts significant anticancer effects at concentrations substantially lower than those proven as effective in monotherapy, suggesting a potential role in dose reduction in conventional agents and, subsequently, mitigation of their adverse effects. Although the enhanced effect of those combinations has been shown in preclinical models, there is a distinct lack of human clinical trials in this matter. Available evidence supports a promising concept of lunasin as a molecular “priming” agent that might complement cytotoxic therapies rather than replace them. This combination-oriented paradigm may represent a shift in lunasin research and offer a novel direction for the use of bioactive peptides in precision oncology; however, further studies exploring this possibility, including human clinical trials, are needed to elucidate lunasin’s role in nutraceutical-assisted cancer therapy.

## 1. Introduction

Cancer is one of the biggest problems for public health, with approximately 20 million new cases and 9.7 million deaths worldwide in 2022. The most common are lung cancer (12.4% of all cancers—2.5 million cases), breast cancer (11.6%—2.3 million cases), and colorectal cancer (9.6%—1.9 million cases). While lung cancer is characterized by the highest mortality rate, the breast and colorectal malignancies emerge as a clinical challenge; breast cancer is the most common cancer diagnosed in women, while colorectal cancer stands as the second leading cause of cancer-related mortality [1].

Breast cancer is a group of malignancies that can be divided into three types, based on molecular and histological profile: hormone-positive, expressing estrogen or progesterone receptor (ER+ or PR+, respectively), expressing human epidermal receptor 2 (HER2+), and triple-negative breast cancer (TNBC (ER−, PR−, HER2−)) [2]. Risk factors of breast cancer include: exogenous hormone exposure, improper diet, lack of physical activity, family history, and genetic predispositions [3]. Most notably, germline mutations in *BRCA1* and *BRCA2* are associated with breast cancer risk. However, mutations in other genes, such as *TP53*, *PTEN*, and *CDH1*, which are all tumor suppressor genes, are also attributed to the risk of this malignancy. *TP53* encodes the p53 protein, which inhibits the cell cycle after DNA damage or hypoxia. Downregulation of *TP53* leads to uncontrolled proliferation of cells. *CDH1* encodes E-cadherin, which is responsible for cell-to-cell adhesion. When the gene is downregulated, epithelial cells might detach from the primary tumor and drive metastasis. Among these, *PTEN* aberrations are particularly relevant, as the loss of *PTEN* function disrupts the PI3K/Akt/mTOR (phosphoinositide 3-kinase/protein kinase B/mechanistic target of rapamycin) signaling pathway, leading to uncontrolled cell proliferation and survival [4].

The development of colorectal cancer is heavily influenced by lifestyle risk factors (high intake of processed foods, alcohol consumption, and smoking), as well as genetic predispositions [5]. Similar to breast cancer, colorectal malignancies are frequently characterized by oncogenic mutations in genes, such as *TP53* or *PIK3CA.* Both those alterations affect the PI3K/Akt pathway. In this malignancy, hyperactivation of this pathway is driven by aberrations not only in *PTEN* but also in the mentioned *PI3KCA* gene, leading to enhanced cell proliferation [6].

In response to the rising global cancer burden, precision medicine and oncology are emerging fields. Although their origins trace back to the 1980s, the most significant breakthrough occurred at the start of the 21st century. The use of bioactive compounds, including peptides, in nutraceutical-assisted cancer therapy offers a promising approach for decreasing the “off-target” toxicity of conventional chemotherapy [7].

Among phytochemicals, lunasin, a soy-derived bioactive peptide, has emerged as a potent anticancer epigenetic agent [8,9]. It was discovered at the end of the 20th century and has since been widely researched, mostly for its antineoplastic effects [10]. This soy peptide inhibits the proliferation and/or growth of cancer cells but does not affect the growth of non-cancerous cells [11]. Lunasin is effective in vitro [9,12,13,14,15,16] and in vivo [17,18]; however, its efficacy has not been consistently supported in clinical trials [19,20]. This effect is presumably caused by the low bioavailability of lunasin in humans, thereby causing a paradox; in vitro studies demonstrate IC50 values of 13–508.6 µM; however, oral administration of this peptide in humans yields systemic concentrations as high as the nanomolar range [21,22].

This review aims to provide a critical synthesis of the possibility of using lunasin in nutraceutical-assisted cancer therapy, instead of as a stand-alone nutraceutical. It may be capable of “priming” cancer cells for enhanced sensitivity to conventional cytotoxic agents by reducing cell resistance or acting synergistically with the drug [7,15,23,24,25,26,27]. This may provide a novel approach to the use of lunasin and other bioactive compounds in combined therapy.

Overcoming chemotherapy resistance with a phytochemical would be a beneficial strategy for patients in oncological care, as it would not give as many side effects as therapy combining two cytotoxic drugs.

Lunasin would not be the first natural compound used in oncology. As for today, few plant-derived pharmaceuticals, i.e., Paclitaxel, camptothecin (Topotecan), and homoharringtonine, have found application in cancer therapeutic strategies and have been approved by the FDA (U.S. Food and Drug Administration) and EMA (European Medicines Agency), as adjuncts in their natural forms or as synthetic analogs as primary therapeutic agents [28,29].

Lunasin’s ability to sensitize cancer cells and enhance the effect of some anticancer drugs, and the application of the FDA-approved natural compounds in cancer support therapy, provide a novel approach to lunasin research.

## 2. Methodology

The literature for this review was searched in electronic databases—PubMed (National Center for Biotechnology Information), Scopus (Elsevier), and Google Scholar, covering publications from 2010 to 2026. The search strategy focused on peer-reviewed studies, clinical trials, and original, experimental papers evaluating the co-exposure to lunasin and conventional drugs in cancer models.

The keywords used for the search included: “lunasin”, “drug combination”, “drug synergy”, “co-exposure”, “breast cancer”, “colon cancer”, “colorectal cancer”.

To ensure a comprehensive literature search and minimize the risk of omitting relevant papers, the initial databases search was supplemented by a semantic search using the AI-assisted research platform Elicit (https://elicit.com) and Scite (https://scite.ai/).

## 3. Structure and Occurrence of Lunasin

### 3.1. Structure

**Figure 1:**
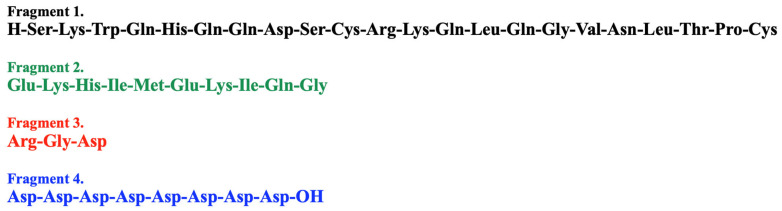
The specific four fragments within the lunasin sequence, based on [31].

Lunasin is a peptide consisting of 43 amino acids. Its molecular weight is estimated at 5.5 kDa [30]. In its structure, four fragments are singled out (Figure 1).

The function of Fragment 1 remains unclear. However, some studies suggest that this fragment, along with Fragment 2, could be responsible for the peptide’s antioxidant and immunomodulatory effects. It may also have an antitransformation effect [30].

Fragment 2 presumably enables peptide attachment to histones, due to its helical structure and structural similarity to chromatin [11].

Fragment 3 is an RGD sequence, consisting of arginine, glycine, and aspartic acid. It is responsible for lunasin attachment to cancer cells, through transmembrane receptors, most significantly integrins—αvβ_3_, αvβ_5_, and α_5_β_1_ [10,11]. This fragment also enables the peptide to reach the nucleus in several hours [30].

Fragment 4 allows lunasin direct attachment to chromatin and binding to deacetylated histones, enabling its epigenetic effect [10,11].

Lunasin’s three-dimensional composition is hard to determine due to its highly disordered structure. Its composition depends on physicochemical conditions and has been determined for 25, 37, 63, 72, 90, and 100 °C after 3 min exposition using circular dichroism [32], and for 37, 47, 57, 67, 77, 87, and 97 °C after 30 min exposition using RP-HPLC (Reverse Phase High-performance Liquid Chromatography) [33]. The unfolding of lunasin starts at 37 °C after 30 min exposition, and increases gradually, reaching 26% degradation at 87 °C [33]. After 3 min of exposure, the degradation starts at 90 °C, reaching 24.4% decrease at 100 °C [32]. In both studies, despite different exposure times, no significant changes in lunasin three-dimensional structure were observed up to ~70 °C, even after longer exposure of 30 min. Lunasin is thermally stable up to 87–90 °C.

### 3.2. Occurrence

Lunasin is found in soy; its amount depends on genetic variability and ranges from 1100 to 14,000 μg per g of extracted soy protein [34,35,36]. It was suggested that it is also present in other cereals, e.g., wheat, rye, barley, and amaranth, but later it was established that they contain “lunasin-like peptides”, with a mechanism of action that is similar to that of lunasin [37].

There is also a new approach to obtaining lunasin, namely, transgenic modifications. Transgenic maize, containing significant amounts of biologically active peptide, was obtained in the amount of 120.34 and 232.51 μg lunasin/g of maize grain in two different modified maize lines overexpressing the *lunasin* gene [38]. It is also possible to obtain transgenic wheat that contains 308.63–436.78 µg lunasin/g of wheat grain. The peptide obtained in this way proved to be biologically active in HT-29 colorectal cancer cells [39].

## 4. Bioavailability of Lunasin

The primary barrier to lunasin reaching the plasma and tissues is the gastrointestinal tract. Like other bioactive peptides, lunasin is susceptible to pepsin activity and the acidic environment of the stomach. Thus, there is a need to establish methods to protect lunasin from being digested in the stomach and allow it to reach the intestines intact, from where it can be absorbed.

The degree of lunasin degradation varies significantly across experimental models. After exposure to pepsin, lunasin-enriched soy flour (27% lunasin) showed rapid degradation after 60 min, and after 180 min, only 25% of its original amount remained intact. Further in vitro digestion with pancreatin resulted in further degradation, reducing intact lunasin to only 3% after 180 min [40]. Naturally occurring protease inhibitors within the soy matrix, such as BBIs (Bowman–Birk protease inhibitors) and KTIs (Kunitz trypsin inhibitors), offer a degree of structural protection. BBIs and KTIs are suggested to protect lunasin mostly from pancreatin digestion. In their presence, 93 and 97% of lunasin remains intact [18]. Similarly, the addition of IBB1 (ratio lunasin: BBI 1:1) to synthetic lunasin increased the intact peptide amount to 34.3% ± 3.7, compared to synthetic lunasin without BBIs, where only 2.6% ± 0.4 of the original amount remained intact [41].

While BBIs and KTIs significantly enhance lunasin resistance to intestinal proteolysis, they remain insufficient to fully protect the peptide from gastric digestion.

Research indicates the mechanism by which lunasin is absorbed in the gastrointestinal tract. To evaluate the intestinal absorption mechanism, the Caco-2 cell line was used. The results indicate that lunasin is absorbed through the Caco-2 cell monolayer, suggesting that lunasin crosses the intestinal epithelial barrier. The main mechanism is suggested to be the paracellular passive pathway via tight junctions [42].

To further elucidate lunasin systemic absorption, research has focused on quantifying lunasin survival and baseline bioavailability across in vivo and human subjects after oral administration. An in vivo study on rats demonstrates high lunasin bioavailability, reaching approximately 30% in the target tissue [18]. However, this high bioavailability has not yet been confirmed in human clinical trials, where the literature remains limited. There are only two studies concentrated on lunasin bioavailability in humans. The first study was conducted on 5 males, aged 18–25 years old, who consumed 50 g of soy protein daily for 5 days. The concentration of lunasin in plasma after 30 min of ingestion was 50.2–110.6 ng/mL (9.98–22 nM), and after 1 h, 33.5–122.7 ng/mL (6.66–24.40 nM). The results indicate bioavailability of lunasin at an average level of 4.5% (2.2–7.8%) [21].

The second evaluation occurred within a triple-blind randomized clinical trial on 31 participants, and an 8-week exposure to 335 mg/d lunasin-enriched soy extract was performed [19,22]. While the peer-reviewed results of this trial demonstrated that the dosage was insufficient to exert a therapeutic effect on the primary cardiometabolic endpoints evaluated [19], preliminary pharmacokinetic data presented in a conference abstract indicated that exposure to 335 mg/d lunasin-enriched soy extract increased plasma levels of lunasin by 44%, reaching 0–10 ng/mL (0–1.99 nM) [22]. Despite the therapeutic neutrality observed, no serious adverse effects were reported. Only isolated, mild gastrointestinal symptoms occurred, including bloating [*n* = 1], abdominal pain [*n* = 1], and difficulty swallowing [*n* = 1] [19]. The lack of significant toxicity supports the safety profile of lunasin for human consumption.

**Figure 2:**
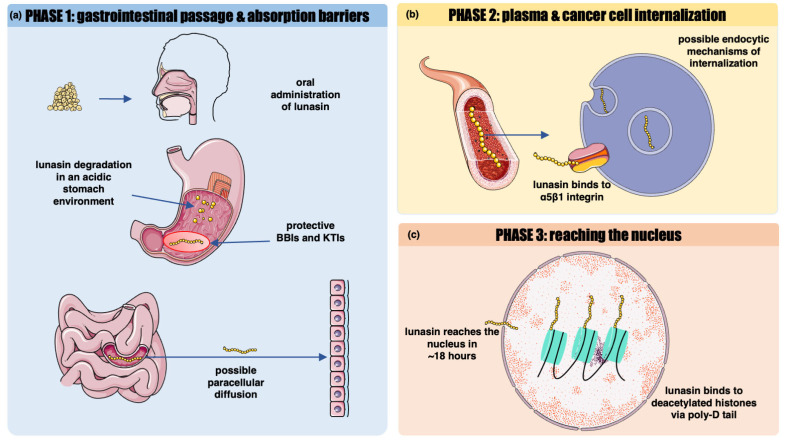
Schematic overview of the pharmacokinetics and bioavailability of lunasin in humans after oral administration. (**a**) Lunasin, administered orally, is extensively digested in the acidic stomach environment. The presence of the BBIs and KTIs provides some protection from digestive enzymes. Lunasin is absorbed in the intestines, presumably by paracellular diffusion. (**b**) Lunasin is detectable in plasma. It binds to αvβ_3_, αvβ_5_, and α_5_β_1_ integrins in the cellular membrane via the RGD motif. Then, presumably, it undergoes endocytosis and enters the cell. (**c**) Lunasin reaches the nucleus in less than 24 h. It binds to deacetylated histones directly via Poly-D-tail (Fragment 4). Image(s) provided by Servier Medical Art (https://smart.servier.com), licensed under CC BY 4.0 (https://creativecommons.org/licenses/by/4.0/ (accessed on 25 June 2026)).

On the cellular level, lunasin is suggested to enter the cells via endocytic mechanisms, involving clathrin-coated vesicles and macropinosomes (Figure 2) [43] in minutes, and reach the nucleus in approximately 18 h [44].

To overcome poor human bioavailability, novel functional encapsulation and structural modification strategies have emerged.

Liposomal encapsulation has been known for about 60 years. However, it was approved for the first time only about 30 years ago. Since then, this method has been a growing trend in nutrition, especially as a delivery system for functional compounds [45].

Lunasin is no different from this trend—novel research is emerging on a more efficient delivery system of this peptide, including in the form of liposomes.

Initial efforts to encapsulate lunasin (beginning in 2021) used amaranth unsaponifiable matter as a peptide carrier. When tested against melanoma cells (B16-F10 and A-375 cell lines), lunasin-loaded liposomes significantly enhanced peptide efficacy, reducing the IC50 by 31.81% and 41.89%, respectively, compared to the same cell lines exposed to non-encapsulated peptide [46]. Liposomes obtained by the same method were also tested against an in vivo model of melanoma. B16-F10 melanoma cells were transplanted into 58 male C57BL/6 mice, aged 4–8 weeks. They were treated daily with lunasin-loaded liposome (15 or 30 mg/kg bw) applied topically or injected subcutaneously for 22 days. Those exposures in vivo proved to increase the level of caspase-3 and increased expression of cell cycle inhibitors, i.e., CDKN2A, CDKN1B, and TP53, resulting in a smaller tumor volume and G1/S phase cell-cycle arrest [47,48].

Liposomes loaded with unsaponifiable amaranth matter are biologically active not only against cancer, but they also show antioxidant activity in vitro in LPS-stimulated (lipopolysaccharide-stimulated) RAW 264.7 macrophages. They decrease ROS (reactive oxygen species) production by 77% at a concentration of 2 mg lunasin/mL [49].

What is important, lunasin, used in the mentioned encapsulation protocol, was obtained from a natural source (soybean flour).

Liposome encapsulation technology is not the only method used to improve lunasin bioavailability. The cyclization technique with recombinant butealse-1 was also used to produce a lunasin variant that is more resistant to digestion. Cyclic lunasin (called by Zhao et al. “clunasin”) showed higher thermostability compared to that generated by non-cyclic lunasin. Authors indicate that at 87 °C, about 26% lunasin was degraded, whereas clunasin was only degraded by approximately 8%. Moreover, clunasin showed higher stability when exposed to digestive enzymes—pepsin and trypsin. After 10 and 20 min, over 90% of non-cyclic lunasin was digested. In contrast, the proportion of clunasin digestion was only 30 and 40%. Clunasin has also shown biological activity and inhibited the growth of HepG2 (hepatocellular carcinoma) cells in an in vitro study [33]. Both the mentioned methods could be used to increase the amount of lunasin reaching the intestines intact and available for absorption.

## 5. Anticancer Effect of Lunasin

Since its discovery, lunasin has been widely studied as a potential anticancer agent. It exerts its anticancer properties through various mechanisms, including epigenetic modifications of histones [50] and inhibition of cell cycle and proliferation via disruption of cancer signaling pathways [7,14,15,16,51].

Lunasin RGD motif not only allows it to enter the cells, but it is also important in its anticancer properties. As mentioned before, lunasin binds to specific integrins αvβ_3_, αvβ_5_, and α_5_β_1_ via the RGD fragment. Those integrins are reportedly overexpressed in breast and colorectal cancers. Through this binding, lunasin disrupts the integrin–extracellular matrix interaction—a mechanism important for angiogenesis, tumor progression, and metastasis. This mechanism of action is indicated in breast cancer, colon cancer, and leukemia [14].

**Figure 3:**
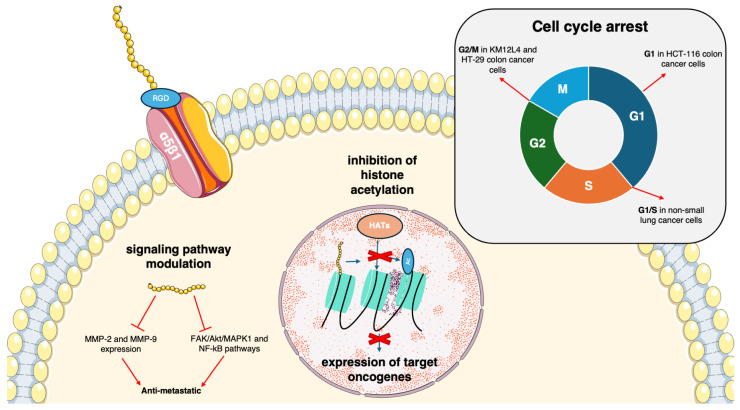
Schematic overview of the anticancer properties of lunasin. Lunasin enters the cell by binding to α_5_β_1_ integrin via RGD motif. It exerts its anti-metastatic properties through decreasing MMP-2 and -9 expression, and inhibition of signaling pathways: FAK/Akt/MAPK1 and NF-kB. After entering the nucleus, lunasin acts as a competitive inhibitor of histone acetyltransferases by directly binding to deacetylated core histones and subsequently blocking their acetylation. It causes transcriptional silencing of target oncogenes. Lunasin also causes cell-cycle arrest in different cancer cell lines—at G2/M in KM12L4 and HT-29 colon cancer cells, at G1 in HCT-116 colon cancer cells, and at G1/S in non-small lung cancer cells. MMP—matrix metalloproteinase, FAK—focal adhesion kinase, Akt—protein kinase B, MAPK1—mitogen-activated protein kinase, NF-kB—nuclear factor kappa-light-chain-enhancer of activated B cells, HATs—histone acetyltransferases. Image(s) provided by Servier Medical Art (https://smart.servier.com), licensed under CC BY 4.0 (https://creativecommons.org/licenses/by/4.0/ (accessed on 25 June 2026)).

Epigenetically, lunasin’s well-established anticancer property is its ability to inhibit histone acetylation. Epigenetic homeostasis is based on the balance between acetylation (performed by HATs (histone acetyltransferases)) and deacetylation (performed by HDACs (histone deacetylases)). Studies indicate that lunasin inhibits HATs by competing with them and subsequently prevents the acetylation of deacetylated core histones (Figure 3). It results in transcriptional silencing of target oncogenes and disruption of cancer development. This may also lead to repression of cell cycle progression [50].

The anti-metastatic and anti-invasive effects of lunasin are suggested to be mediated by decreased expression and activity of MMP-2/-9 (matrix metalloproteinase-2/-9), and suppression of FAK/Akt/MAPK1 (focal adhesion kinase/protein kinase B/mitogen-activated protein kinase) and NF-kB (nuclear factor kappa-light-chain-enhancer of activated B cells) pathways (Figure 3) [14].

Furthermore, lunasin exerts cell line-dependent, phase-specific cell cycle inhibition. It has been demonstrated in non-small-cell lung cancer cells at G1/S phase [51], in HCT-116 colorectal cancer cell line at G1 phase [16], and in another KM12L4 and HT-29 colorectal cancer cell lines at G2/M phase [7,15]. Mechanistically, the modulation of the cell cycle is driven by histone acetylation modifications, suppression of Retinoblastoma protein phosphorylation, and upregulation of CDKN1A and CDKN1B.

The IARC (International Agency for Research on Cancer) report from 2022 highlights three types of cancer as the major problems in today’s public health: lung, breast, and colorectal cancers. Breast cancer is the most common cancer in women and the second most common in the overall population, with 2.3 million new cases. Colorectal cancer, including colon and rectum cancers, is ranked as the third most common cancer in the overall population, reaching 1.9 million new cases. It is the second most deadly cancer, contributing to 900,000 deaths, while breast cancer claims over 660,000 lives [52]. Both breast and colorectal cancers pose a challenge for today’s medicine, and novel therapeutic methods are being sought. Since both breast and colorectal cancer are presumably diet-related [53,54], the use of dietary compounds in chemoprevention is also considered. Lunasin is one of the potential phytochemicals that exerts anticancer and chemopreventive effects against both of those cancers in in vitro and in vivo studies.

### 5.1. Breast Cancer

Lunasin shows anti-proliferative and pro-apoptotic effects on both mildly malignant, ER-positive (MCF-7) and malignant, ER-negative triple-negative breast cancer cells (MDA-MB-231), although it does not affect the growth of normal breast tissue cells (MCF-10A) [12]. Its in vitro anti-proliferative effect was established based on the IC50 value, the concentration that causes 50% inhibition of cell growth.

Different molecular mechanisms have been established for the lunasin effect in these cells. In MCF-7 cells, an in vitro model of hormone-dependent breast cancer, exposure to soy peptide inhibited cell growth, even after their incubation with β-estradiol, which normally stimulates their growth. Moreover, lunasin caused modification in genes encoding inflammation mediators, e.g., IL-6 (interleukin 6), COX-2 (cyclooxygenase-2), and VEGF (vascular endothelial growth factor) [12]. It also increased activity of the *PTEN* promoter and, in consequence, increased concentration of PTEN transcripts and proteins, leading to induction of apoptosis [13].

In MDA-MB-231 cells, lunasin reduced the number of viable cells through inhibition of histone acetylation, disruption of the cyclin D-CDK4/6 axis [9], inhibition of aromatase/ER-α signaling pathway, and disruption of IL-6/VEGF axis [12].

In both MCF-7 and MDA-MB-231, exposure to lunasin caused dose-dependent inhibition of migration and invasion rate. This anti-metastatic effect was caused by the inhibition of FAK and Src phosphorylation, followed by inhibition of FAK-Src complex formation, which has a key role in the metastasis process. Lunasin also caused simultaneous inhibition of PI3K/Akt, MAPK1, and NF-kB pathways through disruption of the integrin signaling axis [14].

Lunasin has also been proven to be effective in an in vivo model—xenograft mice, injected with MDA-MB-231 cells, were treated with lunasin at doses of 4 and 20 mg/kg bw. Both concentrations caused a reduction in tumor incidence and delay in tumor formation (compared to untreated mice) (Table 1 and Table S1) [18].

The range of IC50 values in MCF-7 cells is 232–508 μM and 153–224.7 μM in MDA-MB-231 (Table 1). It is important to mention that all cited studies used synthetic lunasin, that have been suggested to have less pronounced effects compared to naturally derived peptides [16].

### 5.2. Colon Cancer

Lunasin shows anti-proliferative and pro-apoptotic effects in colon cancer cell lines. It reduced cell viability in HCT-116, HT-29, KM12L4, and RKO cells (Table 1). It also induced apoptosis in HCT-116 and KM12L4 cells. Cell-cycle arrest was observed at G1 phase in HCT-116 cells and at G2/M in KM12L4 cells. In HCT-116 cells, lunasin inhibited tumorsphere formation. The most significant growth inhibition effect was observed in KM12L4 cells, as elevated sensitivity to lunasin in those cells is linked to higher expression of α_5_β_1_ integrin [15].

Lunasin exposure reduced Bcl-2 protein expression, increased Bax protein expression, and contributed to the formation of caspase-3 expression; those changes trigger the mitochondrial pathway and cytochrome c/caspase-3 signaling cascade, leading to cell apoptosis [15].

Interestingly, HCT-116 cells exhibit a significantly higher IC50 value when treated with synthetic lunasin, compared to the peptide purified from defatted soybean flour. It is indicated that synthetic lunasin differs in secondary and tertiary structures compared to the naturally derived peptide. Higher inhibitory potential of natural lunasin may also be due to other natural compounds present in natural extracts, protecting peptides from degradation [16].

An in vivo study conducted on mice showed that lunasin has different efficacy depending on the way of administration. The peptide administered intraperitoneally (4 mg/kg bw) was more effective in liver metastasis inhibition than orally gavaged lunasin, although it was administered in higher concentrations (8 and 20 mg/kg bw). Intraperitoneal injections of lunasin also caused inhibition of histone acetylation, suggesting an epigenetic mechanism of liver metastasis reduction. After oral administration, increased acetylation of histones H3 and H4 was observed (but non-significant in H3 in a concentration of 8 mg/kg bw) and differences are presumably caused by diverse routes of lunasin administration [17].

## 6. Lunasin–Conventional Drug Combination

**Figure 4:**
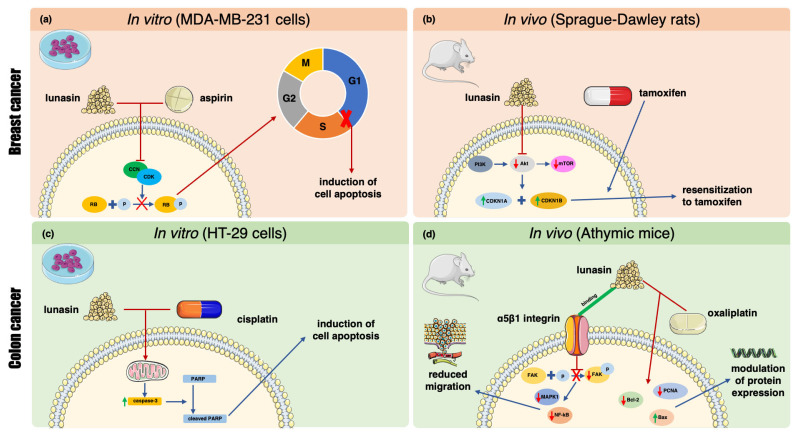
Schematic overview of the multi-targeted molecular pathways modulated by lunasin in combination with conventional therapeutic agents. (**a**) In an in vitro MDA-MB-231 cell line model, combination of lunasin and aspirin enhances modulation of the CCN/CDK/RB pathway, inhibiting RB phosphorylation and inducing S-phase arrest and apoptosis. (**b**) In an in vivo Sprague-Dawley rat model, lunasin overcomes tamoxifen resistance by inhibiting the PI3K/Akt/mTOR pathway and upregulating CDKN1A and CDKN1B. (**c**) In an in vitro colon cancer HT-29 cell line model, lunasin enhances cisplatin-induced cytotoxicity through activation of caspase-3 and an increase in PARP cleavage. (**d**) In an in vivo athymic mouse model, lunasin binds directly to α5β1 integrin, suppressing the FAK/MAPK1/NF-kB signaling cascade, causing reduced migration. A combination of lunasin with oxaliplatin results in the downregulation of PCNA and Bcl-2 with upregulation of Bax, causing modulation of protein expression. CCN—cyclins, CDK—cyclin-dependent kinases, RB—Retinoblastoma protein, PI3K—Phosphoinositide 3-kinase, Akt—protein kinase B, mTOR—mechanistic target of rapamycin, CDKN1A—cyclin-dependent kinase inhibitor 1A, CDKN1B—cyclin-dependent kinase inhibitor 1B, PARP—Poly(ADP-ribose)polymerase, FAK—focal adhesion kinase, MAPK1—mitogen-activated protein kinase 1, NF-kB—nuclear factor kappa-light-chain-enhancer of activated B cells, PCNA—Proliferating Cell Nuclear Antigen. Image(s) provided by Servier Medical Art (https://smart.servier.com), licensed under CC BY 4.0 (https://creativecommons.org/licenses/by/4.0/ (accessed on 25 June 2026)).

Breast and colon cancers are among the most common malignancies, and therapies for those diseases are still being improved, e.g., because of chemotherapy resistance of cancer cells. Conventional drugs are effective. Yet, they cause many adverse effects, e.g., nausea, vomiting, weakness, pain, and depression [55,56,57]. It may be beneficial for cancer therapy to pair conventional drugs with natural compounds that are easy to administer and may act synergistically with chemotherapy. Lunasin is one of the candidates that could be used in adjuvant cancer therapy. It shows anticancer effects on its own; however, concentrations that are effective in vitro are difficult to obtain in humans by regular consumption. Studies suggest that by oral administration, lunasin can reach a concentration of the nanomolar range, which is much lower than doses used in in vitro studies (Table 1). Thus, it might be beneficial to combine conventional anticancer drugs with lunasin, instead of using it as a stand-alone nutraceutical (Table 2 and Table 3 and Table S2). Most publications on lunasin-drug combinations are from 2010/11; however, Kusmardi et al. revisited this idea in 2021 and 2025, proposing a new direction in lunasin research (Figure 4).

For approximate recalculation of doses (in vivo doses to humans), the HED (Human Equivalent Dose) formulation was used to compare administered doses to therapeutic doses in humans [58].

### 6.1. Breast Cancer

Lunasin was combined with aspirin in vitro in MDA-MB-231 cells, representing an in vitro model of breast cancer. This combination proved to be effective in cell growth inhibition—concentrations of 10 µM lunasin + 0.5 mM aspirin caused a decrease in cell viability by 54%, while 10 µM + 2 mM caused 73% reduction in cell number. A 10 µM + 2 mM combination of lunasin + aspirin also caused cell-cycle arrest in S phase, and subsequently apoptosis, resulting in a higher percentage of apoptotic cells (both in early and late stages) compared to both compounds used alone. Cell-cycle arrest and induction of apoptosis are presumably caused by the enhanced effect of the modulation of expression of genes involved in cell growth control (e.g., CCN/CDK/CDKN2A/RB pathway and PI3K/AKT pathway) [27].

Tamoxifen is a selective estrogen receptor modulator and is FDA-approved for the treatment of breast cancer. In breast tissue, it binds to estrogen receptors, competing with estrogen, resulting in an antitumor effect. It also slows down the cell cycle, making it a cytostatic drug. Typical dosage of tamoxifen used in breast cancer is 20–40 mg per day (~0.3–0.6 mg/kg bw) [57].

An in vivo study was conducted on female Sprague-Dawley rats aged 6 weeks induced with breast cancer by DMBA (7,12-Dimethylbenz[a]anthracene)—rats received treatment of lunasin (500 mg/kg bw; which corresponds to concentration of approximately 1.42 mM in blood), tamoxifen (10 mg/kg bw ~1.61 mg/kg dose for human) or a combination of both for 8 weeks after breast cancer was induced and the tumor reached the size of 1–2 cm^3^. The results suggest that lunasin in combination caused significant upregulation of CDKN1A expression compared to tamoxifen alone and no treatment, with levels of CDKN1A being higher only in the healthy group. This effect is presumably caused by the reduction in tamoxifen resistance—it is linked to lower levels of CDKN1A and CDKN1B. Lunasin causes upregulation of CDKN1A and CDKN1B expression, reversing the resistance mechanism. Moreover, lunasin may downregulate Akt, a part of the PI3K/Akt/mTOR pathway, whose activation is also linked to tamoxifen resistance in breast cancer. That being said, lunasin presumably decreases tamoxifen resistance, which could be the mechanism of enhanced therapeutic response of this combination [23]. Notably, this study used concentrations of lunasin higher than the typical dietary exposure threshold—while it limits the study’s relevance to standard nutritional intake of this peptide, it provides an interesting mechanism for its enhancement of tamoxifen effects. In the same model (although rats were aged 4–6 weeks) with the same lunasin concentrations, after exposure to a combination, a reduction in ICAM-1 (Intercellular Adhesion Molecule 1) and an increase in Cadherin1 (epithelial cadherin) levels were observed—both molecules are involved in breast cancer adhesion and progression, and ICAM-1 may also be involved in the metastasis process [59]. In the adjuvant group, the observed changes were not significantly different compared to tamoxifen alone, suggesting that the obtained effect might be contributed by the drug alone and not the combination; however, lunasin administered alone was able to reduce ICAM-1 expression while increasing E-Cadherin expression, although to a lesser extent than tamoxifen [24].

### 6.2. Colon Cancer

Cisplatin and oxaliplatin are platinum-based antineoplastic drugs, classified as cytotoxics. Cisplatin is approved by the FDA (the U.S. Food and Drug Administration) as treatment for ovarian, testicular, and bladder cancers. It has also been successfully used off-label in breast, cervical, and endometrial cancers, as well as some gastrointestinal malignancies, e.g., gastric and hepatobiliary. Its mechanism of action is based on disrupting DNA strands by covalently binding platinum to guanine and adenine. Typical dosage of cisplatin is 20–100 mg/m^2^ (approx. 0.54–2.7 mg/kg) [55]. Oxaliplatin is FDA-approved for the treatment of stage III colorectal cancer and metastatic colorectal cancer. Platinum in the drug binds to the DNA. It causes inhibition of replication, transcription, and cell-cycle arrest and leads to cell death. Typical dosage of oxaliplatin is 85 mg/m^2^ (approx. 2.1–2.3 mg/kg) [56].

Lunasin in combination with cisplatin was used in HT29 cells—an in vitro model of colon cancer. As mentioned above, cisplatin is not typically used in colon cancer, and it is not FDA-approved for therapy in this type of malignancy. Moreover, studies suggest that cisplatin shows no activity against colorectal cancer cells. Colon cancer cells’ cisplatin resistance may be explained by alterations in DNA damage-induced TP53 signaling [60]. Some studies succeeded in overcoming this resistance by combining cisplatin with other compounds, e.g., aspirin; this combination resulted in enhancement of cisplatin activity against colon cancer through suppression of anti-apoptotic Bcl-2, suppression of PI3K/Akt, and an increase in PARP1 cleavage and Bax upregulation [61]. Lunasin (30 and 60 µM) combination with cisplatin at concentrations of 0.1, 1, 10, and 50 µM resulted in an enhanced effect. To compare it to doses used in cancer therapy, it is approximately 0.4, 4.1, 38.9, and 194.2 mg/m^2^, respectively. All of the combined doses resulted in a significant decrease in proliferation compared to both lunasin and cisplatin alone. Exposure to the highest concentrations of cisplatin and peptide combination resulted in 98.6% cell viability decrease, while 50 µM of cisplatin alone decreased cell viability by approximately 30%. Moreover, caspase-3 level was significantly increased upon exposure to a combination of cisplatin (50 µM) and lunasin (50 µM), and this effect was more pronounced (2220 mU/mL) compared to both substances used alone (1137 mU/mL and 310 mU/mL, respectively). These results suggest an enhancement of combination of lunasin and cisplatin [7]. Although the biological mechanism of this combination is promising, its clinical relevance in colorectal cancer remains limited, since cisplatin is not FDA-approved for this type of malignancy. However, future studies might focus on evaluating a combined approach in cisplatin-approved cancer types, since the results are encouraging.

Another chemotherapeutic whose effect is enhanced by lunasin is oxaliplatin. As mentioned, oxaliplatin is the standard course of therapy in advanced and metastatic colorectal cancer. A study was conducted on 37 male athymic mice aged 7 weeks. KM12L4 colon cancer cells were injected into the spleen. A combination of lunasin with oxaliplatin was administered by intraperitoneal injection (IP), in doses of 4 mg/kg bw lunasin daily (which corresponds to a maximum theoretical concentration in blood of approximately 6.64 µM) + 5 mg/kg bw (~0.8 mg/bw dose for humans) oxaliplatin twice per week. This combination reduced liver metastatic nodules in mice injected with KM12L4 colon cancer cells. Lunasin caused a reduction in liver metastatic nodules from 28 to 14, and the combination of lunasin with the drug increased the reduction to 5. The anti-metastatic combined effect is explained by lunasin binding to α5β1 integrin, directly, and therefore suppression of FAK/MAPK1/NF-κB signaling pathway, resulting in reduced migration of cancer. Tumor burden (defined as tumor weight over body mass) was decreased from 0.13 in the control to 0.10 in the sample with lunasin, and the combination caused further reduction to 0.04. A combination of lunasin with oxaliplatin significantly reduced the expression of PCNA (Proliferating Cell Nuclear Antigen) by 86% compared to the control group. A combination of lunasin with oxaliplatin caused significantly higher reduction in expression of anti-apoptotic Bcl-2 compared to control and lunasin or oxaliplatin alone. Pro-apoptotic protein Bax was more induced by the combination compared to both agents alone. The enhanced effect of those two compounds is dual: the combination potentiates the protein expression modulation (reduction in anti-apoptotic Bcl-2 and upregulation of pro-apoptotic Bax) and a complementary effect, as lunasin acts as an anti-metastatic agent and oxaliplatin acts mostly as a cytotoxic agent; however, a specific mechanism has not been provided [25].

As mentioned above, lunasin enhancement of cisplatin effect may be caused by resensitization of cells to this drug. Some studies suggest that lunasin is capable of changing colon cancer cells’ response to cisplatin through molecular alterations. Similarly, in combination with oxaliplatin, lunasin may reduce cancer cells’ oxaliplatin resistance. Oxaliplatin resistance may be caused by the deregulation of the PI3K/Akt signaling pathway [62]. Lunasin is a potential PI3K/Akt pathway inhibitor, which could presumably lower the oxaliplatin resistance and sensitize cells to oxaliplatin. Inhibition of PI3K/Akt may be responsible for the observed reduction in anti-apoptotic Bcl-2. The described effect was more pronounced in the combination group compared to both agents alone, which suggests synergy. Doses of the drug injected intraperitoneally used in in vivo models are difficult to recalculate in comparison to therapeutic doses in mg/m^2^ or µM used in vitro due to the specific distribution path of the drug after intraperitoneal administration. The compound, having been administered intraperitoneally, is absorbed into the bloodstream through the portal vein. Absorption through the portal vein results in the first-pass effect, because before the compound reaches the bloodstream, it might be eliminated by hepatic detox processes. This type of administration is presumably better for lunasin, because it bypasses the gastrointestinal tract and avoids digestion. However, there is no data on its first-pass effect. Hence, we cannot determine the final concentration achieved in blood [63].

### 6.3. Inhibitory Potential of Lunasin in Chemical Induction of Cancer

Although this study was not performed on breast or colon cancer models, but on mouse fibroblasts (NIH/3T3), it is important to mention, as it indicates that lunasin enhanced the chemopreventive effect of aspirin. In an in vitro model of mouse, fibroblasts (NIH/3T3 cells induced with chemical carcinogens: DMBA and MCA (methylcholanthrene)), the addition of 1 µM lunasin to 2 mM aspirin caused a 1.5-fold increase in cell growth inhibition effect. An amount of 1 µM lunasin in combination with 0.5 and 2 mM aspirin also significantly reduced foci formation [26]. Hsieh et. al. suggest that the mechanism of the observed effect was similar to the one described in their in vitro study [27].

## 7. Discussion

Lunasin has been widely researched as a potential anticancer agent. Despite being effective in in vitro and in vivo studies, human clinical trials did not confirm those results. However, clinical data on lunasin are very limited, and some of them lack results. The unsatisfactory effects of lunasin exposure in human clinical trials are most probably caused by its limited bioavailability after oral administration.

The novel possible use of lunasin might be nutraceutical-assisted cancer therapy. As data suggest, the addition of lunasin to other anticancer drugs might improve their efficacy, resulting in lowering the dose of chemotherapeutics. Consequently, patients might benefit from it, as it might lead to fewer side effects. Although the preclinical studies are very promising, there needs to be further investigations on this topic, including human clinical trials, to establish if it might be used in cancer treatment in the future.

Lunasin, in combination with tamoxifen, exerts enhanced effects in vivo [23]. As mentioned, tamoxifen is an estrogen receptor modulator FDA-approved for breast cancer therapy. Although tamoxifen is characterized by relatively low toxicity [64], its efficacy is limited by the development of tamoxifen resistance in breast cancer patients [65]. The tamoxifen resistance is driven by various mechanisms, including, e.g., the overexpression of HDAC1, and high expression of *p*-Akt. Both those mechanisms are related to the activation of the PI3K/Akt/mTOR pathway. HDAC1 is a part of the cascade activating the PI3K/Akt pathway, and high expression of *p*-Akt is a part of the PI3K/Akt pathway that is associated with activation of this cascade. To summarize, the PI3K/Akt/mTOR signaling pathway is an important target for nullifying the tamoxifen resistance. As it is indicated in the studies, lunasin downregulates the Akt, resulting in downregulation of the PI3K/Akt/mTOR pathway. Studies suggest that the current direction of nullifying tamoxifen resistance may be achieved by the use of Akt inhibitors, e.g., gefitinib [65]. Gefitinib is an inhibitor of epidermal growth factor receptor tyrosine kinase, FDA-approved for the treatment of non-small-cell lung cancer [66]. Like many drugs used in cancer treatment, gefitinib has burdensome side effects, such as rash, diarrhea, nausea, and vomiting [67]. Results of existing studies are promising, as lunasin in co-exposure with tamoxifen enhanced the drug’s effect in an in vivo model by downregulation of Akt, and presumably resensitizing cells to tamoxifen. This mechanism needs to be further examined, as it is promising, but the evidence is limited. However, it might be the direction of future studies on lunasin as an adjunct therapy in breast cancer treated with tamoxifen.

A combined effect of aspirin and lunasin might be attributed to cell-cycle arrest via inhibition of the CCN/CDK/RB pathway in an in vitro model of TNBC [27]. TNBC is a particular clinical challenge, as patients frequently develop resistance to chemotherapy, and TNBC itself is the most lethal and most aggressive subtype of breast cancer. Possible mechanisms of chemoresistance in TNBC are, e.g., avoidance of apoptosis via Bcl-2 pro-survival protein expression and upregulation of PI3K/Akt/mTOR pathway [68]. Studies indicate that combined exposure to lunasin and aspirin results in inhibition of RB phosphorylation and subsequent cell-cycle arrest. The CCN/CDK complex formation is affected by Akt, further affecting the RB phosphorylation and cell cycle progression [69]. It is not clear how the aspirin and lunasin combination mechanism works exactly. However, as lunasin is the inhibitor of Akt, we may suspect that the observed changes are the result of PI3K/Akt/mTOR pathway inhibition. Those changes were more pronounced in combination, compared to aspirin and lunasin alone. As the PI3K/Akt/mTOR cascade is indicated to participate in TNBC chemoresistance, this effect might be clinically relevant in TNBC therapies. However, as it is only an assumption and only the CCN/CDKK/RB pathway has been examined, further research is needed to expand the scope of research on the mechanism related to that combination.

The treatment of colorectal cancer depends on the stage of illness progression. In early stages (stage I), it is treated with resection, in stages II–III, referred to as locally advanced surgical resection with optional complementary chemotherapy. In stage IV, also referred to as metastatic or advanced, systemic chemotherapy is used. Chemotherapeutics utilized in colorectal cancer include: fluoropyrimidine (5-fluorouracil, capecitabine) and oxaliplatin. Oxaliplatin is a third-generation platinum-based drug whose anticancer mechanism is based on binding to DNA bases and subsequently inhibiting DNA synthesis, and is usually used in the advanced stages of colorectal cancer [70]. However, platinum-based drugs have many side effects, including long-term toxicity, which is related to heavy metal exposure. Studies indicate that platinum-based chemotherapy leads to systemic platinum accumulation in the human body, resulting in long-term side effects. What is more, platinum-based chemotherapeutics, although effective against cancer cells, lack selectivity, which may cause acute side effects (acute kidney injury, myelosuppression, gastrointestinal toxicity). Long-term toxicity includes: nephrotoxicity, neurotoxicity, ototoxicity, and immunotoxicity [71]. Considering the platinum-based chemotherapy consequences, seeking methods to lower the dose of this type of chemotherapy without losing its efficacy is vital. Research indicates that lunasin might be a possible candidate as an adjunct to primary chemotherapy with oxaliplatin. In an in vivo study, lunasin in combination with oxaliplatin showed a dual mechanism of action. On one hand, lunasin reduced the metastasis rate via direct binding to integrin α5β1 and subsequent inhibition of phosphorylation of FAK. It caused downregulation of MAPK1 and NF-kB, which are involved in the metastasis of colorectal cancer. This effect was confirmed by a decrease in tumor burden and a lower number of liver metastatic nodules. On the other hand, lunasin enhanced the effect of oxaliplatin. Combined exposure exerted a better effect in the modulation of expression of proteins: pro-apoptotic Bax increase, and anti-apoptotic Bcl-2 decrease. Lunasin and oxaliplatin combination also resulted in a reduction in PCNA expression [25]. Research indicates that higher expression of PCNA is related to higher liver metastasis in colorectal cancer [72]. Both those results combined provide a promising direction for future studies on that combination. Lunasin, in addition to oxaliplatin treatment, might enable lowering the platinum-based chemotherapeutic dose and, subsequently, protect the patients from severe long-term side effects.

Cisplatin is another platinum-based drug. As mentioned, it is not FDA-approved for colon cancer treatment. However, it is used in other solid tumors treatment, and it also exerts similar short- and long-term side effects, such as those of oxaliplatin. Therefore, it is important to explore the possibility of lowering its dose by adding an adjunct, without a decrease in therapy efficacy. In an in vitro study, Dia et al. indicate that lunasin in combination with cisplatin resulted in upregulation of caspase-3, inducing the apoptosis of colorectal cancer cells [7]. As cisplatin is not FDA-approved for this type of malignancy, the clinical relevance of this study is limited. However, cisplatin, which is not approved for colorectal cancer treatment, and oxaliplatin, FDA-approved for this malignancy, have a similar course of action in colorectal cancer cells. Research indicates that both cisplatin and oxaliplatin cause upregulation of caspase-3 [73,74]. As lunasin caused an enhanced effect on HT-29 colorectal cancer cells in combination with cisplatin, we may suspect that it might exert a similar enhancement with oxaliplatin, as both drugs share a similar mechanism regarding caspase-3. This study might also encourage further studies on lunasin overcoming cisplatin resistance in its FDA-approved malignancies.

Although the current studies support the hypothesis that a lunasin–conventional drug combination might be effective in chemoprevention by, e.g., alleviating chemoresistance of cancers, the evidence is limited by a lack of clinical human trials. They are needed to further elucidate lunasin’s potential role in nutraceutical-assisted cancer therapy. However, lunasin bioavailability is the bottleneck for performing those studies. While human clinical trials for lunasin have been conducted in other therapeutic areas—such as a 12-month pilot trial for Amyotrophic Lateral Sclerosis (ALS) [20] and aforementioned 8-week study on cardiometabolic risk factors [19]—they were unsuccessful in demonstrating clinical efficacy, primarily because oral administration failed to provide the necessary systemic peptide concentrations for lunasin to exert its therapeutic potential.

For further investigation of lunasin’s role as an adjunct in cancer treatment, the development of novel systems for improving lunasin bioavailability after oral ingestion by humans is needed. Without the novel delivery systems for this peptide, proving that lunasin might be effective and useful in cancer therapy will be limited, as it is hard to achieve desired lunasin concentrations (effective in vitro and in vivo) in human plasma after oral administration. This is the biggest limitation of studies on all bioactive peptides; however, as novel delivery systems are being explored, further investigations in this topic will be needed, provided effective delivery of lunasin to plasma.

## 8. Conclusions

Lunasin is a promising anticancer and antioxidant agent that has been proven to be effective in in vitro and in vivo studies. However, while lunasin possesses robust anti-tumor activity, its monotherapeutic IC50 values (13–508.6 µM) significantly exceed the concentrations achieved through standard oral administrations, with bioavailability of only 4.5% that results in approximately nanomolar lunasin concentrations in plasma.

Thus far, only two clinical trials with lunasin were performed, but the obtained results do not indicate any significant changes in the analyzed parameters—presumably due to the low bioavailability of lunasin and the inability to achieve therapeutic doses established in in vitro studies. In a triple-blind clinical trial, 31 participants (19 females, 12 males, aged 61 ± 9.9 years) were exposed for 8 weeks to 335 mg lunasin-enriched soy extract per day. However, it was not specified how much lunasin was administered. This exposure proved to be ineffective in reducing cardiometabolic risk factors, i.e., serum lipids, glucose, insulin resistance, blood pressure, BMI (Body Mass Index), or waist circumference [19]. Another clinical trial on 50 participants (29 males and 21 females, mean age 60 years) with a diagnosis of ALS (Amyotrophic Lateral Sclerosis) was exposed to lunasin (Lunarich X Capsules, Provantage and Reliv Now—6 capsules per day, 1 scoop twice per day, 1.5 scoops twice per day, respectively). After a year of intervention, no significant difference in histone acetylation levels was observed [20]. Analysis of intraperitoneal in vivo models suggests that by bypassing the gastrointestinal tract, lunasin proves to be effective in vivo, and in clinical trials, an effective concentration of lunasin in plasma was not achievable.

Lunasin shows many potential mechanisms of enhancement of drug effect: (I) reducing drug resistance through molecular alterations (e.g., inhibition of PI3/Akt signaling pathway, upregulation of CDKN1A and CDKN1B), (II) complementary anti-metastatic action to cytotoxic drug, (III) cell-cycle arrest (cancer cells are more sensitive to apoptosis signaling), and (IV) synergistic upregulation of pro-apoptotic molecules (e.g., caspase-3, Bax).

For now, lunasin is only available in soy-based food products and in the form of diet supplements. Clinical trials that were supposed to approve lunasin for cancer therapy have been conducted, but were unsuccessful in achieving therapeutic effects, presumably because of bioavailability barriers. However, as mentioned above, several natural compounds have been successfully applied in cancer therapy and approved by the FDA and/or EMA. Mansuori et al., in their review, highlight a few of them approved in the therapy of breast cancer, such as: Paclitaxel and Docetaxel (*Taxus* species), Vincristine and Vinblastine (*Catharanthus roseus*), Topotecan and Irinotecan (*Camptotheca acuminata*) [28]. Of particular relevance to lunasin research is homoharringtonine, originating from *Cephalotaxus*. Homoharringtonine and its semi-synthetic analog (omacetaxine mepesuccinate) have been approved by the FDA for the treatment of CML (chronic myeloid leukemia), in cases where two or more TKIs failed [75]. Importantly, homoharringtonine is administered by subcutaneous injections, thereby bypassing the whole gastrointestinal tract and avoiding enzymatic degradation associated with oral delivery. The examples above demonstrate that naturally derived compounds can be successfully used in modern cancer therapy and highlight the potential of the alternative routes of administration for natural compounds or their semisynthetic analogs. Homoharringtonine history may indicate a novel path in lunasin research, especially when taking different administration methods into consideration.

Lunasins’ ability to disrupt α5β1 integrin signaling and therefore FAK/MAPK1/NF-κB signaling pathway or PI3K/AKT survival pathway enables “prime” malignant cells or reduces drug resistance, and thereby lowers the dose of conventional chemotherapy drugs. A reduced number of administered chemotherapy doses, whose effectiveness is still unchanged, could be beneficial to patients, as the side effects of conventional chemotherapy may be bothersome. As precision medicine develops, considering natural compounds in combination with conventional chemotherapy that result in enhanced therapeutic response and at the same time do no harm to normal cells might be a novel method to alleviate some of the most troubling side effects and improve cancer treatment.

## Figures and Tables

**Table 1:** The studies with lunasin in in vitro and in vivo models of breast and colon cancers: lunasin exposure versus control (without lunasin).

Cancer type	Type of Study	Model	Lunasin Origin	IC50	Concentration of Lunasin	Time of Exposure	Effect	Mechanism	Reference
Breast cancer	In vitro	MDA-MB-231 cells	Synthetic, purity > 95% (Kaijie Peptide Company, Chengdu, China)	153 µM * (48 h)	25–200 µM	24–72 h	Cell growth inhibition (dose-dependent manner, decreased number of viable cells by ~25–65%)	Suppression of the aromatase/ERα signaling pathway and disrupting the IL-6/VEGF-mediated microenvironment, triggering pro-apoptotic signaling	[12]
					5 µM	24–48 h	No significant changes in inflammatory mediators and number of dead cells		
					50 µM	24–48 h	Decreased *COX-2* mRNA levels (0.31-fold change) Increased IL-6 levels (0.3-fold change) Increased number of early-stage apoptotic cells (1.37-fold change) and total number of apoptotic cells (1.29-fold change)		
			Synthetic (Chengdu KaiJie Bio-Pharmaceutical (Chengdu, China))	181 µM *	10–200 µM	48 h	Decreased number of viable cells by ~10–55% (dose-dependent manner)	Histone H3 (Lys 9, Lys 14) and H4 (Lys 5, 8, 12, 16) acetylation inhibition Downregulation of cyclin D1, cyclin D3, CDK4, CDK6	[9]
			Synthetic, purity > 95% (GL Biochem (Shanghai, China))	224.7 µM * (24 h) 194.9 µM * (48 h)	10–320 µM	24–48 h	Decreased number of viable cells by ~15–65%	Inhibition of FAK/Src phosphorylation by lunasin, disruption of the integrin-mediated signaling axis, leading to the simultaneous suppression of PI3K/Akt, MAPK1, and NF-κB pathways	[14]
					10–20 µM	12 h	Decreased migration by ~75–85%		
						24 h	Decreased migration (by ~80–90%) and invasion (by ~75–80%), reduced activity and expression of MMP-9, and MMP-2		
		MCF-7 cells	Synthetic, purity > 95% (Kaijie Peptide Company, Chengdu, China)	232 µM *	5–200 µM	24–72 h	Cell growth inhibition (dose-dependent manner, decreased number of viable cells by ~20–45%)	Suppression of the aromatase/ERα signaling pathway and disruption of the IL-6/VEGF-mediated microenvironment, leading to pro-apoptotic signaling	[12]
					5 µM	24–48 h	No significant changes in inflammatory mediators		
					50 µM	24 h	Increased *COX-2* mRNA levels (1.29-fold change)		
						48 h	Increased number of early-stage apoptotic cells (1.34-fold change) and total number of apoptotic cells (1.08-fold change)		
			Synthetic, purity > 95% (GL Biochem (Shanghai, China))	508.6 µM * (24 h) 431.9 µM (48 h)	40–320 µM	24–48 h	Decreased number of viable cells by ~15–45%	Downregulation of MMP-9 Inhibition of phosphorylation of FAK and Src, followed by disruption of PI3K/Akt and FAK/Akt/MAPK1 and NF-κB pathways	[14]
					10–20 µM	12 h	Decreased migration (by ~50–55%)		
						24 h	Decreased migration (by ~70%) and invasion (by ~60%), reduced activity and expression of MMP-9, no effect on MMP-2		
			Synthetic, unknown purity (American Peptide Co., Sunnyside, CA, USA)	n/a *	50 nM	24 h	No significant effect on PTEN expression Increased number of apoptotic cells (13-fold change)	PTEN upregulation and PTEN-mediated apoptosis	[13]
					2 µM	24 h	Increased number of apoptotic cells (21-fold change)		
	In vivo	32 athymic NCr-nu/nu mice, aged 6 weeks, with MDA-MB-231 cells (1 × 10^7^) implanted subcutaneously	Synthetic lunasin (Chengdu KaiJie Bio-Pharmaceutical Co., Chengdu, China)	n/a *	20 mg/kg bw (intraperitoneal injections, 3 times a week)	2 months	57% lower tumor incidence compared to control group (without lunasin) Reduction in tumor size by 23%	Apoptosis and necrosis induction in MDA-MB-232 cells, inhibition of cell proliferation	[18]
					4 mg/kg bw (intraperitoneal injections, 3 times a week)		43% lower tumor incidence compared to control group (without lunasin) Reduction in tumor size by 34%		
Colon cancer	In vitro	HCT-116 cells	Purified from defatted soybean flour, purity > 90%	26.3 µM	1–100 µM	24 h	Decreased number of viable cells (up to ~70% at 100 µM)	Mitochondrial pathway-mediated effects, Bcl-2 downregulation on protein level, Bax upregulation on protein level, and the subsequent cytochrome c/caspase-3 signaling cascade	[15]
			Synthetic, purity > 95% (Chengdu KaiJie Biopharm Co., Ltd. (Chengdu, China))	107.5 ± 1.9 µM *	5 µM	72 h	No significant effect on proliferation	Caspase-3 activation, decreased PARP1 protein levels, increased CDKN1A protein expression levels	[16]
					10–160 µM		Decreased number of viable cells (~15–60%)		
					5–10 µM	10 days	No significant effect on tumorsphere formation		
					20–160 µM		Inhibition of tumorsphere formation (~20–50%)		
					20–80 µM	72 h	Apoptosis induction, increased number of total apoptotic cells (1.3–1.8-fold change) Cell-cycle arrest at G1 phase		
		HCT-116 OxR cells	Purified from defatted soybean flour, purity > 90%	31. 6 µM	1–100 µM	24 h	Decreased number of viable cells (up to ~80% at 100 µM)	Mitochondrial pathway-mediated effects, Bcl-2 downregulation on protein level, Bax upregulation on protein level, and the subsequent cytochrome c/caspase-3 signaling cascade	[15]
		HT-29 cells		61.7 µM	1–100 µM	24 h	Decreased number of viable cells (up to ~60% at 100 µM)		
		HT-29 OxR cells		n/a	1–100 µM	24 h	No significant effect on cell viability up to 50 µM concentration		
		KM12L4 cells		13 µM	1–100 µM	24 h	Decreased number of viable cells (~20% at 1 µM, up to ~90% at 50 µM)		
					5 µM		G2/M cell-cycle arrest Increased number of apoptotic cells (1.6-fold change)		
					10 µM		G2/M cell-cycle arrest Upregulation of CDKN1A (2.2-fold change) and CKDN1B (2.3-fold change) Increased number of apoptotic cells (1.82-fold change)		
					25 µM		Increased number of apoptotic cells (2-fold change)		
		KM12L4 OxR cells		34.7 µM	1–100 µM	24 h	Decreased cell viability (up to ~80% at 100 µM)		
					10 µM		Increase in caspase-3 expression (1.7-fold change)		
					25 µM		Upregulation of CDKN1A and CDKN1B		
		RKO cells		21.6 µM	1–100 µM	24 h	Decreased cell viability (up to ~90% at 100 µM)		
		RKO OxR cells		38.9 µM	1–100 µM	24 h	Decreased cell viability (up to ~65% at 100 µM)		
	In vivo	Mice, aged 6–8 weeks, injected with KM12L4 cells (1 × 10^6^) into the spleen	Isolated and purified from defatted soybean flour, purity > 95%	n/a	4 mg/kg bw (intraperitoneal injections)	28 days	Reduction in liver metastasis by 50% (compared to untreated group) Liver weight/body weight ratio reduction by 23% (compared to untreated group) Decreased acetylation of H3 and H4 histones	Inhibition of p300 enzyme activity, leading to reduction in histone acetylation	[17]
					8 mg/kg bw (gavage)	28 days	Reduction in number of liver metastases by 56% (compared to untreated group) Increased acetylation of histone H4 (2.3-fold)		
					20 mg/kg bw (oral gavage)	28 days	Reduction in number of liver metastases by 94% (compared to untreated group) Increased acetylation of histones H3 (3.3-fold) and H4 (2.7-fold)		

* synthetic lunasin.

**Table 2:** Comparison of lunasin combinations with conventional drugs and their effects—in vitro studies.

Type of Cancer	Model	Lunasin Origin	Time of Exposure	Drug	Concentration of Lunasin	Concentration of Drug	Effect	Mechanism of Effect	Reference
Breast cancer	MDA-MB-231 cells	Synthetic, purity > 95% (American Peptide Co., Sunnyvale, CA, USA)	48–72 h	Aspirin (IC50 = 1.7 mM)	0 µM	0.5–2.5 mM	Decreased cell number by ~15–95%	Cell-cycle arrest and induction of apoptosis presumably caused by synergistic modulation of expression of genes involved in cell growth control (e.g., CCN/CDK/CDKN2A/RB pathway and PI3K/AKT pathway)	[27]
					1 µM	0.5–2.5 mM	No significant difference from aspirin only		
					10 µM	0.5–2.5 mM	Decreased cell number by ~30–95% S-phase cell-cycle arrest Increased number of early and late-stage apoptotic cells		
					25 µM				
Colon cancer	HT-29 cells	Purified from defatted soybean flour, purity ~90%	24 h	Cisplatin (IC50 = 76.7 µM)	0–60 µM	0.1–50 µM	Decreased cell viability by ~10–98.6%	Enhancement of cisplatin-induced caspase-3 activation and growth inhibition	[7]
					50 µM	0 µM	Caspase-3 activity 1137 mU/mL (increased compared to untreated cells 6.5-fold)		
						1–50 µM	Caspase-3 activity increased 2-fold compared to cisplatin only (compared to untreated cells: 12.6-fold)		
	KM12L4 cells	Purified from defatted soybean flour, purity > 90%	24 h	Oxaliplatin	0–25 µM	0.5 µM	No decrease in cell viability	Restoring Bax/Bcl-2 balance	[15]
						0.5–2 µM	Decreased cell viability by ~10–90%		
Inhibitory effect on cancer induction	NIH/3T3 (mouse fibroblast cells) cancer induced with DMBA	Synthetic, purity > 95% (American Peptide Co., Sunnyvale, CA, USA)	24–72 h	Aspirin	0 µM	2000 µM	40% reduction in cell number compared to untreated cells	Prevention of chemical carcinogen-induced transformation of fibroblasts Modulation of expression of genes involved in cell growth control	[26]
					1 µM *	25 µM	No reduction in cell number compared to untreated cells No effect on foci formation		
						125 µM	20% reduction in cell number compared to untreated cells Non-significant reduction in foci formed		
						500–2000 µM	30–60% reduction in cell number compared to untreated cells Reduction in foci formed ~1.75–7-fold		
	NIH/3T3 (mouse fibroblast cells) cancer induced with MCA		24–72 h	Aspirin	0 µM	2000 µM	40% reduction in cell number compared to untreated cells		
					1 µM *	25 µM	No reduction in cell number compared to untreated cells No effect on foci formation		
						125 µM	20% reduction in cell number compared to untreated cells non-significant reduction in foci formed		
						500–2000 µM	25–60% reduction in cell number compared to untreated cells Reduction in foci formed ~2.2–11-fold		

* synthetic lunasin.

**Table 3:** Comparison of lunasin combinations with conventional drugs and their effects—in vivo studies.

Type of Cancer	Model	Lunasin Origin	Time of Exposure	Drug	Concentrations of Lunasin	Concentration of Drug	Effect	Mechanism of Effect	Reference
Breast cancer	30 female Sprague-Dawley rats aged 6 weeks induced with breast cancer by DMBA	Extracted from soybean of the Grobogan variety (Indonesian Legumes and Tubers Crops Research Institute (Balitkabi), Malang, East Java)	8 weeks after the tumor reached 1–2 cm^3^	Tamoxifen	500 mg/kg bw ~1.42 mM	10 mg/kg bw	Upregulation of CDKN1A protein—1.23-fold increase compared to rats with cancer receiving no treatment and 1.13-fold increase compared to rats receiving only tamoxifen	Nullifying tamoxifen resistance by upregulation of CDKN1A and presumably downregulation of Akt, part of PI3K/Akt/mTOR pathway	[23]
	24 female Sprague-Dawley rats aged 4–6 weeks induced with breast cancer by DMBA		8 weeks after the tumor reached 1–2 cm^3^		500 mg/kg bw ~1.42 mM	10 mg/kg bw	Significant decrease in ICAM-1 and increase in Cadherin-1 levels, but statistically not significant compared to Tamoxifen alone	No significant effect of lunasin addition to tamoxifen	[24]
Colorectal cancer	37 male athymic mice aged 7 weeks injected with KM12L4 colon cancer cells (into spleen)	Purified from defatted soybean flour, ≥90% purity	28 days	Oxaliplatin	4 mg/kg bw daily ~6.64 µM (IP)	5 mg/kg bw (~15 mg/m^2^) twice per week (IP)	Reduction in liver metastatic nodules 5.6-fold compared to mice receiving no treatment (control group) and 2.8-fold compared to lunasin alone Tumor burden decreased 3.25-fold compared to control group, 2.5-fold compared to lunasin alone and 2-fold compared to oxaliplatin alone PCNA expression was decreased 7.14-fold compared to control group, 4.86-fold compared to lunasin alone and 2.86-fold compared to oxaliplatin alone Bcl-2 expression was decreased 1.75-fold compared to control group, 1.56-fold compared to lunasin alone and 1.33-fold compared to oxaliplatin alone	Suppression of FAK/MAPK1/NF-κB signaling pathway Combination potentiates the protein expression modulation (reduction in anti-apoptotic Bcl-2 and upregulation of pro-apoptotic Bax) and complementary effect, as lunasin acts as anti-metastatic agent and oxaliplatin acts mostly as cytotoxic agent; however, a specific mechanism has not been provided	[25]

## Data Availability

No new data were created or analyzed in this study. Data sharing is not applicable to this article.

## Supplementary Materials

The following supporting information can be downloaded at: https://www.mdpi.com/article/10.3390/ijms27136079/s1.

## Abbreviations

The following abbreviations are used in this manuscript:

TNBC	Triple Negative Breast Cancer
PI3K	Phosphoinositide 3-kinase
Akt	Protein kinase B
mTOR	Mechanistic target of rapamycin
FDA	U.S. Food and Drug Administration
EMA	European Medicines Agency
BBI	Bowman–Birk protease Inhibitor
KTI	Kunitz trypsin inhibitor
LPS	Lipopolysaccharide
ROS	Reactive oxygen species
HATs	Histone acetyltransferases
HDACs	Histone deacetylases
MMP	Matrix metalloproteinases
FAK	Focal adhesion kinase
MAPK1	Mitogen-activated protein kinase
NF-κB	Nuclear factor kappa-light-chain-enhancer of activated B cells
IARC	International Agency for Research on Cancer
IL-6	Interleukin-6
COX-2	Cyclooxygenase-2
VEGF	Vascular endothelial growth factor
PTEN	Phosphatase and tensin homolog deleted on chromosome ten
ER-α	Estrogen receptor alpha
HED	Human equivalent dose
DMBA	7,12-Dimethylbenz[a]anthracene
ICAM-1	Intercellular Adhesion Molecule 1
PARP	Poly(ADP-ribose)polymerase
IP	Intraperitoneal
PCNA	Proliferating Cell Nuclear Antigen
MCA	3-Methylcholantrene
BMI	Body Mass Index
ALS	Amyotrophic Lateral Sclerosis
