# Supplementary material for: Co-Exposure to Lunasin and Other Drugs as a Potential Chemopreventive Strategy Against Breast and Colon Cancers: A Review
Source: Int J Mol Sci. 2026 Jul 7;27(13):6079. doi: 10.3390/ijms27136079 (PMC13360953; doi:10.3390/ijms27136079)
Supplement: Supplementary file 1 [file ijms-27-06079-s001.zip › Supplementary Material - Table S2.pdf]

Table S2 Comparison of lunasin combinations with conventional drugs and their effects – in vitro studies.

Type of cancer	Model	Lunasin origin	Time of exposure	Drug	Concentration of lunasin	Concentration of drug	Effect	Mechanism of effect	Reference
**Breast cancer**	MDA-MB-231 cells	Synthetic, purity >95% (American Peptide Co., Sunnyvale, CA, USA)	48 – 72 h	Aspirin (IC50 = 1,7 mM)	0 μM	0.5 mM	Decreased cell number by ~15 – 95%	Cell cycle arrest and induction of apoptosis presumably caused by synergistic modulation of expression of genes involved in cell growth control (e.g. CCN/CDK/CDKN2A/RB pathway and PI3K/AKT pathway	[1]
						1 mM			
						2 mM			
						2.5 mM			
					1 μM	0.5 mM	No significant difference from aspirin only		
						1 mM			
						2 mM			
						2.5 mM			
					10 μM	0.5 mM	Decreased cell number by approx. 30 %		
						1 mM			
						2 mM	most significant difference (73% compared to 54% aspirin only – 1.35-fold increase) S-phase cell cycle arrest Increased number of early and late stage apoptotic cells (by 7 and		

							12%, respectively, compared to aspirin alone)		
						2.5 mM	Decreased cell number by ~95 %		
					25 µM	0.5 mM	Decreased cell number by ~30 – 95%		
						1 mM			
						2 mM			
						2.5 mM			
**Colon cancer**	HT-29 cells	Purified from defatted soybean flour, purity ~90%	24 h	Cisplatin (IC50 = 76,7 µM)	0 µM	0.1 µM	Decreased cell viability by ~10%	Synergistic potentiation of cisplatin-induced caspase-3 activation and growth inhibition	[2]
						1 µM	Decreased cell viability by ~15%		
						10 µM	Decreased cell viability by ~20%		
						50 µM	Decreased cell viability by ~30%		
					30 µM	0,1 µM	Decreased cell viability by ~80%		
						1 µM	Decreased cell viability by ~85%		
						10 µM	Decreased cell viability by ~90%		

					50 µM	Decreased cell viability by ~90%		
				60 µM	0.1 µM	Decreased cell viability by ~85%		
					1 µM	Decreased cell viability by ~90%		
					10 µM	Decreased cell viability by ~95%		
					50 µM	Decreased cell viability by 98,6%		
				50 µM	0 µM	Caspase-3 activity 1137 mU/ml (increased compared to untreated cells 6.5-fold)		
					1 µM<br>10 µM<br>50 µM	Caspase-3 activity increased 2-fold compared to cisplatin only (compared to untreated cells: 12.6-fold)		
KM12L4 cells	Purified from	24 h	Oxaliplatin	0 µM	0.5 µM	No decrease in cell viability	Restoring Bax/Bcl-2 balance	[3]

		defatted soybean flour, purity >90%				1 μM	Decreased cell viability by ~10%		
						2 μM	Decreased cell viability by ~20%		
					10 μM	0.5 μM	Decreased cell viability by ~50%		
						1 μM	Decreased cell viability by ~60%		
						2 μM	Decreased cell viability by ~60%		
					25 μM	0.5 μM	Decreased cell viability by ~60%		
						1 μM	Decreased cell viability by ~90%		
						2 μM	Decreased cell viability by ~90%		
**Cancer induction**	NIH/3T3 (mouse fibroblast cells) cancer induced with DMBA	Synthetic, purity >95% (American Peptide Co., Sunnyvale, CA, USA)	24-72 h	Aspirin	0 μM	2000 μM	40% reduction in cell number compared to untreated cells	Prevention of chemical carcinogens-induced transformation of fibroblasts	[4]
					1 μM*	25 μM	no reduction in cell number compared to untreated cells	Modulation of expression of genes involved in cell growth control	

						No effect on foci formation
					125 μM	20% reduction in cell number compared to untreated cells Non-significant reduction of foci formed
					500 μM	30% reduction in cell number compared to untreated cells Reduction of foci formed ~1.75-fold
					2000 μM	60% reduction in cell number compared to untreated cells Reduction of foci formed ~7-fold
NIH/3T3 (mouse fibroblast cells) cancer induced with MCA		24-72 h	Aspirin	0 μM	2000 μM	40% reduction in cell number compared to untreated cells
				1 μM*	25 μM	No reduction in cell number compared to untreated cells No effect on foci formation

	125 μM	20% reduction in cell number compared to untreated cells non-significant reduction of foci formed	
	500 μM	25% reduction in cell number compared to untreated cells Reduction of foci formed ~2.2-fold	
	2000 μM	60% reduction in cell number compared to untreated cells Reduction of foci formed ~11-fold	

1. Hsieh, C.-C.; Hernández-Ledesma, B.; de Lumen, B.O. Lunasin, a Novel Seed Peptide, Sensitizes Human Breast Cancer MDA-MB-231 Cells to Aspirin-Arrested Cell Cycle and Induced Apoptosis. *Chemico-Biological Interactions* **2010**, *186*, 127–134, doi:10.1016/j.cbi.2010.04.027.
2. Dia, V.P.; Mejia, E.G.D. Lunasin Promotes Apoptosis in Human Colon Cancer Cells by Mitochondrial Pathway Activation and Induction of Nuclear Clusterin Expression. *Cancer Letters* **2010**, *295*, 44–53, doi:10.1016/j.canlet.2010.02.010.
3. Dia, V.P.; de Mejia, E.G. Lunasin Induces Apoptosis and Modifies the Expression of Genes Associated with Extracellular Matrix and Cell Adhesion in Human Metastatic Colon Cancer Cells. *Molecular Nutrition & Food Research* **2011**, *55*, 623–634, doi:10.1002/mnfr.201000419.
4. Hsieh, C.-C.; Hernández-Ledesma, B.; de Lumen, B.O. Lunasin–Aspirin Combination Against NIH/3T3 Cells Transformation Induced by Chemical Carcinogens. *Plant Foods Hum Nutr* **2011**, *66*, 107–113, doi:10.1007/s11130-011-0229-1.
