# Supplementary material for: Co-Exposure to Lunasin and Other Drugs as a Potential Chemopreventive Strategy Against Breast and Colon Cancers: A Review
Source: Int J Mol Sci. 2026 Jul 7;27(13):6079. doi: 10.3390/ijms27136079 (PMC13360953; doi:10.3390/ijms27136079)
Supplement: Supplementary file 1 [file ijms-27-06079-s001.zip › Supplementary materials Table S1.pdf]

Table S1 The studies with lunasin in in vitro and in vivo models of breast and colon cancers: lunasin exposure versus control (without lunasin).

Cancer type	Type of study	Model	Lunasin origin	IC50	Concentration of lunasin	Time of exposure	Effect	Mechanism	Reference
Breast cancer	In vitro	MDA-MB-231 cells	Synthetic, purity >95% (Kaijie Peptide Company, Chengdu, China)	153 µM* (48h)	25-200 µM	24 h	Cell growth inhibition (dose-dependent manner, decreased number of viable cells by ~25-65%)	Suppression of the aromatase/ERα signaling pathway and disrupting the IL-6/VEGF-mediated microenvironment, triggering pro-apoptotic signaling	[1]
						48 h			
						72 h			
					5 µM	24 h	No significant changes in inflammatory mediators and number of dead cells		
						48 h			
					50 µM	24 h	Decreased *COX-2* mRNA levels (0.31-fold change) Increased IL-6 levels (0.3-fold change)		
						48 h	Increased number of early-stage apoptotic cells (1.37-fold change) and total number of apoptotic cells (1.29-fold change)		

			Synthetic (Chengdu KaiJie Bio-Pharmaceutical (Chengdu, P. R. China))	181 µM*	10 µM	48 h	Decreased number of viable cells by ~10%	Histone H3 (Lys 9, Lys 14) and H4 (Lys 5, 8, 12, 16) acetylation inhibition<br>Down-regulation of cyclin D1, cyclin D3, CDK4, CDK6	[2]
					50 µM		Decreased number of viable cells by ~20%		
					100 µM		Decreased number of viable cells by ~30%		
					200 µM		Decreased number of viable cells by ~55% (dose-dependent manner)		
			Synthetic, purity >95% (GL Biochem (Shanghai, China))	224,7 µM * (24 h)<br>194,9 µM * (48 h)	40-320 µM	24 h	Decreased number of viable cells by ~15-65%	Inhibition of FAK/Src phosphorylation by lunasin, disruption of the integrin-mediated signaling axis, leading to the simultaneous suppression of PI3K/Akt, MAPK1, and NF-κB pathways	[3]
					10-320 µM	48 h	Decreased number of viable cells by ~20-60%		
					10 µM	12 h	Decreased migration (by ~ 75%)		
						24 h	Decreased migration (by ~80%) and invasion (by ~75%), reduced activity and expression of MMP-9, and MMP-2		

					20 µM	12 h	Decreased migration (by ~ 85%)		
						24 h	Decreased migration (by ~ 90%) and invasion (by ~ 80%), reduced activity and expression of MMP-9, and MMP-2		
		MCF-7 cells	Synthetic, purity >95% (Kaijie Peptide Company, Chengdu, China)	232 µM*	25-200 µM	24 h	Cell growth inhibition (dose-dependent manner, decreased number of viable cells by ~20-30%)	Suppression of the aromatase/ERα signaling pathway and disruption the IL-6/VEGF-mediated microenvironment, leading to pro-apoptotic signaling	[1]
					5-200 µM	48 h	Cell growth inhibition (dose-dependent manner, decreased number of viable cells by ~25-45%)		
						72 h			
					5 µM	24 h	No significant changes in inflammatory mediators		
						48 h			
					50 µM	24 h	Increased *COX-2* mRNA levels (1.29-fold change)		

						48 h	Increased number of early-stage apoptotic cells (1.34-fold change) and total number of apoptotic cells (1.08-fold change)		
			Synthetic, purity >95% (GL Biochem (Shanghai, China))	508.6 μM * (24h) 431.9 μM (48 h)	40-320 μM	24 h 48 h	Decreased number of viable cells by ~15-45%	Downregulation of MMP-9 Inhibition of phosphorylation of FAK and Src, followed by disruption of PI3K/Akt and FAK/Akt/MAPK1 and NF-κB pathways	[3]
					10 μM	12 h	Decreased migration (by ~ 55%)		
						24 h	Decreased migration (by ~ 70%) and invasion (by ~ 60%), reduced activity and expression of MMP-9, no effect on MMP-2		
					20 μM	12 h	Decreased migration (by ~50%)		
						24 h	Decreased migration (by ~ 70%) and invasion (by ~ 60%), reduced the activity and expression of MMP-9, no effect on MMP-2		

			Synthetic, unknown purity (American Peptide Co., Sunnyside, CA, USA)	n/a*	50 nM	24 h	No significant effect on PTEN expression<br>Increased number of apoptotic cells (13-fold change)	PTEN upregulation and PTEN-mediated apoptosis	[4]
					2 μM	24 h	Increased *PTEN* transcript levels, promoter activity and PTEN protein level<br>Increased number of apoptotic cells (21-fold change)		
	In vivo	32 athymic NCr-nu/nu mice, aged 6 weeks, with MDA-MB-231 cells (1x10⁷) implanted subcutaneously	Synthetic lunasin (Chengdu KaiJie Bio-Pharmaceutical Co., Chengdu, P.R. China)	n/a*	20 mg/kg bw (intraperitoneal injections, 3 times a week)	2 months	57% lower tumor incidence compared to control group (without lunasin)<br>Reduction in tumor size by 23%	Apoptosis and necrosis induction in MDA-MB-232 cells, inhibition of cell proliferation	[5]
					4 mg/kg bw (intraperitoneal injections, 3 times a week)		43% lower tumor incidence compared to control group (without lunasin)<br>Reduction in tumor size by 34%		
Colon cancer	In vitro	HCT-116 cells	Purified from defatted soybean flour, purity >90%	26.3 μM	1 – 100 μM	24 h	Decreased number of viable cells (up to ~ 70% at 100 μM)	Mitochondrial pathway-mediated effects, Bcl-2 downregulation on protein level, Bax upregulation on protein	[6]

								level, and the subsequent cytochrome c/caspase-3 signaling cascade	
			Synthetic, purity >95% (Chengdu KaiJie Biopharm Co., Ltd. (Chengdu, China))	107.5 ± 1.9 µM *	5 µM	72 h	No significant effect on proliferation	Caspase-3 activation, decreased PARP1 protein levels, increased CDKN1A protein expression levels	[7]
					10 – 160 µM		Decreased number of viable cells (~15-60%)		
					5-10 µM	10 days	No significant effect on tumorsphere-formation		
					20 – 160 µM		Inhibition of tumorsphere-formation (~20-50%)		
					20 µM	72 h	Apoptosis induction, increased number of total apoptotic cells (1.3-fold change) Cell-cycle arrest at G1 phase		
					40 µM		Apoptosis induction, increased number of total apoptotic cells (1.7-fold change) Cell-cycle arrest at G1 phase		

					80 μM		Apoptosis induction, increased number of total apoptotic cells (1.8-fold change) Cell-cycle arrest at G1 phase		
		HCT-116 OxR cells	Purified from defatted soybean flour, purity >90%	31. 6 μM	1 – 100 μM	24 h	Decreased number of viable cells (up to ~ 80% at 100 μM)	Mitochondrial pathway-mediated effects, Bcl-2 downregulation on protein level, Bax upregulation on protein level, and the subsequent cytochrome c/caspase-3 signaling cascade	[6]
		HT-29 cells		61.7 μM	1 – 100 μM	24 h	Decreased number of viable cells (up to ~ 60% at 100 μM)		
		HT-29 OxR cells		n/a	1 – 100 μM	24 h	No significant effect on cell viability up to 50 μM concentration		
		KM12L4 cells		13 μM	1 – 100 μM	24 h	Decreased number of viable cells (~20% at 1 μM, up to ~90% at 50 μM)		
					5 μM		G2/M cell cycle arrest		

							Increased number of apoptotic cells (1.6-fold change)		
					10 µM		G2/M cell cycle arrest<br>Upregulation of CDKN1A (2.2-fold change) and CKDN1B (2.3-fold change)<br>Increased number of apoptotic cells (1.82-fold change)<br>Bax protein upregulation (2.2-fold change)<br>Bcl-2 protein downregulation (2-fold change)<br>Increase in caspase-3 expression (1.8-fold change)		
					25 µM		Increased number of apoptotic cells (2-fold change)		
		KM12L4 OxR cells		34.7 µM	1 – 100 µM	24 h	Decreased cell viability (up to ~80% at 100 µM)		

					10 μM		Bax protein upregulation (2.3-fold change)<br>Bcl-2 protein downregulation (1.5-fold change)<br>Increase in caspase-3 expression (1.7-fold change)		
					25 μM		Upregulation of CDKN1A and CDKN1B		
		RKO cells		21.6 μM	1 – 100 μM	24 h	Decreased cell viability (up to ~90% at 100 μM)		
		RKO OxR cells		38.9 μM	1 – 100 μM	24 h	Decreased cell viability (up to ~ 65% at 100 μM)		
	In vivo	Mice, aged 6-8 weeks, injected with KM12L4 cells (1x10^6) into the spleen	isolated and purified from defatted soybean flour, purity >95%	n/a	4 mg/kg bw (intraperitoneal injections)	28 days	Reduction in liver metastasis by 50% (compared to untreated group)<br>Liver weight/body weight ration reduction by 23%	Inhibition of p300 enzyme activity, leading to reduction in histone acetylation	[8]

							(compared to untreated group) Decreased acetylation of H3 and H4 histones		
					8 mg/kg bw (gavage)	28 days	Reduction in number of liver metastasis by 56% (compared to untreated group) Non-significant liver weight/body weight ration reduction No significant effect on H3 acetylation, increased acetylation of histone H4 (2.3-fold)		
					20 mg/kg bw (oral gavage)	28 days	Reduction in number of liver metastasis by 94% (compared to untreated group) Non-significant liver weight/body weight ration reduction Increased acetylation of histones H3 (3.3-fold) and H4 (2.7-fold)		

1. Hsieh, C.-C.; Wu, C.-H.; Peng, S.-H.; Chang, C.-H. Seed-Derived Peptide Lunasin Suppressed Breast Cancer Cell Growth by Regulating Inflammatory Mediators, Aromatase, and Estrogen Receptors. *Food Nutr Res* **2023**, *67*, 10.29219/fnr.v67.8991, doi:10.29219/fnr.v67.8991.
2. Hernández-Ledesma, B.; Hsieh, C.-C.; de Lumen, B.O. Relationship between Lunasin's Sequence and Its Inhibitory Activity of Histones H3 and H4 Acetylation. *Mol Nutr Food Res* **2011**, *55*, 989–998, doi:10.1002/mnfr.201000632.
3. Jiang, Q.; Pan, Y.; Cheng, Y.; Li, H.; Liu, D.; Li, H. Lunasin Suppresses the Migration and Invasion of Breast Cancer Cells by Inhibiting Matrix Metalloproteinase-2/-9 via the FAK/Akt/ERK and NF-κB Signaling Pathways. *Oncology Reports* **2016**, *36*, 253–262, doi:10.3892/or.2016.4798.
4. Pabona, J.M.P.; Dave, B.; Su, Y.; Montales, M.T.E.; de Lumen, B.O.; de Mejia, E.G.; Rahal, O.M.; Simmen, R.C.M. The Soybean Peptide Lunasin Promotes Apoptosis of Mammary Epithelial Cells via Induction of Tumor Suppressor PTEN: Similarities and Distinct Actions from Soy Isoflavone Genistein. *Genes Nutr* **2013**, *8*, 79–90, doi:10.1007/s12263-012-0307-5.
5. Hsieh, C.-C.; Hernández-Ledesma, B.; Jeong, H.J.; Park, J.H.; Lumen, B.O. de Complementary Roles in Cancer Prevention: Protease Inhibitor Makes the Cancer Preventive Peptide Lunasin Bioavailable. *PLOS ONE* **2010**, *5*, e8890, doi:10.1371/journal.pone.0008890.
6. Dia, V.P.; de Mejia, E.G. Lunasin Induces Apoptosis and Modifies the Expression of Genes Associated with Extracellular Matrix and Cell Adhesion in Human Metastatic Colon Cancer Cells. *Molecular Nutrition & Food Research* **2011**, *55*, 623–634, doi:10.1002/mnfr.201000419.
7. Fernández-Tomé, S.; Xu, F.; Han, Y.; Hernández-Ledesma, B.; Xiao, H. Inhibitory Effects of Peptide Lunasin in Colorectal Cancer HCT-116 Cells and Their Tumorsphere-Derived Subpopulation. *Int J Mol Sci* **2020**, *21*, 537, doi:10.3390/ijms21020537.
8. Dia, V.P.; Mejia, E.G. de Potential of Lunasin Orally-Administered in Comparison to Intraperitoneal Injection to Inhibit Colon Cancer Metastasis in Vivo. *Journal of Cancer Therapy* **2013**, *4*, 34–43, doi:10.4236/jct.2013.46A2005.
